# Viral transduction for T cell engineering in immunotherapy

**DOI:** 10.1016/j.mocell.2026.100311

**Published:** 2026-01-04

**Authors:** Yikhyeon Seo, Jimin Pak, Jiyun Han, Joonbeom Bae, Soo Seok Hwang

**Affiliations:** 1 School of Biological Sciences, Seoul National University, Gwanak-ro 1, Seoul 08826, Republic of Korea; 2 Institute of Molecular Biology and Genetics, Seoul National University, Gwanak-ro 1, Seoul 08826, Republic of Korea; 3 Department of Biotechnology, College of Life Sciences and Biotechnology, Korea University, Seoul 02841, Republic of Korea

**Keywords:** CAR-T, Immunotherapy, Lentiviral transduction, Retroviral transduction, T cell engineering

## Abstract

Viral transduction of primary T cells enables stable genetic engineering for research and immunotherapy, supporting both transgene overexpression and gene deletion. Although the overall workflow can be similar to transduction in other mammalian cell lines, primary T cell culture imposes distinct requirements such as cell-state-dependent nuances shaped by T cell activation and proliferation, which can make it challenging to obtain a sufficient number of genetically engineered T cells. This article provides practical guidance for researchers new to T cells but familiar with basic mammalian cell culture.

## INTRODUCTION

Genetic modification of primary T cells using viral vectors has transformed both experimental immunology and clinical therapy. Retroviral and lentiviral systems remain the mainstay, supporting stable integration and broad construct expression. However, successful application requires careful attention to vector design, timing relative to T cell activation, and optimization of culture conditions (Sadelain et al., 2017). This MiniResource complements the preceding article on T cell isolation and activation, extending the workflow into stable genetic engineering. Here, we provide concise guidance on viral vector preparation, transduction strategies, and troubleshooting to help researchers generate reproducible populations of engineered T cells for diverse experimental and therapeutic objectives.

## MAIN BODY

### Preparation of Virus

It is essential to select the most suitable viral platform based on the purpose of the research. Retroviral and lentiviral vectors are widely used for T cell engineering, each with distinct properties (Table 1). Efficient transduction depends on T cell activation, as retroviruses require cell division (Cepko and Pear, 2001), while lentiviruses can transduce non-dividing cells (Ramezani and Hawley, 2002, Schaffer et al., 2008). Retroviral vectors are preferred in preclinical mouse models to lentiviral vectors because of restricted lentiviral infection in primary murine T cells (Baumann et al., 2004, Kerkar et al., 2011). In contrast, lentiviral vectors offer higher efficiency, higher transgene capacity, and safer integration profiles, making them suitable for diverse applications.

**Table 1:** Comparison of retroviral and lentiviral platforms for T cell engineering

Feature	Retroviral vectors	Lentiviral vectors
Infection targets	Activated T cells (mitosis-required)	Both dividing and non-dividing T cells
Integration profile	Favors transcription start sites and regulatory regions	Favors intron and intergenic regions
Transgene capacity	About 8 kb	About 10 kb
Tropism (envelope)	VSV-G or specific (ecotropic or amphotropic)	VSV-G or other pseudotypes possible
Typical use in T cells	Common in preclinical mouse models; cost-effective; strong expression	Widely used for chimeric antigen receptor T (CAR-T) cells or TCR-T cells; flexible timing, larger inserts
Advantages	Efficient in early blasts; Retronectin synergy; Cost-effective	Works across wider activation windows; consistent transduction across diverse donors
Disadvantages	Narrow timing; mitosis-dependent; risk of insertional mutagenesis	Complex packaging; higher cost; risk of biosafety by wide tropism

Viral particles are usually produced using HEK293T-based cell lines. In standard systems, the transfer plasmids are co-transfected with packaging and envelope plasmids, whereas helper-free lines such as Plat-E or Phoenix only require the transfer plasmid (Morita et al., 2000, Swift et al., 2001). Frozen cell line stocks of low passages are thawed in advance. For optimal recovery, cells are seeded in the desired plate at 70%-80% confluency before transfection (Brown et al., 2020). Transfection step introduces plasmid DNA (deoxyribonucleic acid) into cells. Common transfection methods include polyethylenimine and lipid-based reagents such as Lipofectamine, both widely used for efficient virus production (Kichler, 2004, Pear, 2001, Hammill et al., 2016; Kichler, 2004; Pear, 2001). Researchers may adjust DNA–reagent ratios to optimize transfection. Viral particles in culture supernatant can be collected 2-3 days after transfection. Cell debris from the producing cell line should be removed by centrifugation and filtration. Stocks are snap-frozen and stored at −80 °C. Stock aliquots for single use are recommended to avoid a decrease in viral titer by thawing and refreezing. It can be considered to concentrate the viral supernatant for higher transduction efficiency.

### T Cell Preparation for Transduction

Selecting proper T cells to transduce is the first strategic decision. In clinical settings, many workflows transduce bulk peripheral T cells from peripheral blood mononuclear cells, which is a standard in the clinical manufacturing setting (Labbe et al., 2021). In contrast, using immunophenotypically defined subsets like murine CD62L⁺CD44⁻ naïve T cells from secondary lymphoid organs enhances experimental consistency and reproducibility in mouse-based basic research.

A key constraint is that resting T cells (memory and especially naïve) are quiescent, making them intrinsically poor recipients for gene delivery (Roe et al., 1993, Verhoeyen et al., 2009). Therefore, it is crucial to activate T cells using the essential signals: TCR ligation (Signal 1), co-stimulation (Signal 2), and cytokines to drive differentiation (Signal 3). Here, we primarily assume activation of murine naïve T cells on anti-CD3 coated plate (with anti-CD28 provided plate-bound or soluble) and encourage researchers to add Signal-3 cytokines that match their purpose of the experiment (e.g., Th1/Th2/Th17/iTreg polarization). T cells begin proliferating ∼24 hours post-stimulation (Iezzi et al., 1998), then rapidly expand (24-72 hours), providing an optimal window for efficient transduction (Lanitis et al., 2021, Rosselle et al., 2025) and scalable culture from 96-well plates to flasks.

### Viral Transduction of Primary T Cells

Efficient transduction can be enhanced by spinoculation (centrifugal inoculation), which increases virus-cell contact (O'doherty et al., 2000). Polycations such as polybrene reduce charge repulsion (Davis et al., 2004), and retronectin further improves efficiency by bridging viral particles and T cells (Rajabzadeh et al., 2021, Tonks et al., 2005). In practice, activated T cell cultures are typically centrifuged at 300-500 g for 2-5 minutes to pellet the cells, after which the supernatant is carefully removed to avoid dislodging the pellet. Viral supernatant supplemented with polybrene (4-10 μg/mL) is then added to each well, and spinoculation is performed at 300-1000 g for 60-120 minutes at 32 °C.

### After Transduction: Expansion, Readouts, and Troubleshooting Guides

After viral exposure, the inoculum can be replaced with cytokine-supplemented medium and cultures can remain on the same anti–CD3/CD28–coated surface with additional cytokines to steer differentiation as needed. In routine maintenance, primary T cells can be kept between ∼0.5−2.0 × 10^6^ cells/mL, refresh medium every 2-3 days, and split back to avoid nutrient depletion and crowding.

Transgene expression typically becomes robust by 48-72 hours after transduction. Reporters can be co-expressed to index successful gene delivery—fluorescent proteins such as GFP, tdTomato and surface markers such as tNGFR (truncated NGFR) or Thy1.1 (CD90.1), which can be quantified by flow cytometry (percent positive and mean fluorescence intensity, MFI). When transduction efficiency is low, it is often attributable to an insufficient multiplicity of infection (MOI) or suboptimal viral titer. In such cases, quantitative polymerase chain reaction (qPCR)-based titration of viral stocks (or integrated copies) provides a validated measure of viral titer, and virus concentration steps (eg, polyethylene glycol or ultracentrifugation) can be used to increase MOI without excessive volume. Common issues include polycation toxicity (Kurachi et al., 2017; Lin et al., 2011; Shifrut et al., 2018), insufficient activation (Schwartz, 2003), or overstimulation leading to poor survival or function (Vardhana et al., 2020). Once T cells are primed, the proliferation program can proceed without direct TCR/co-stimulatory signals. Cytokines provide support during culture; IL-2 is commonly used for CD8^+^ T cell survival and expansion, with IL-7 or IL-15 as alternatives. Practical solutions are summarized in Table 2.

**Table 2:** Troubleshooting common issues in viral transduction

Problem	Likely cause	Quick fixes
Low % positive cells	Insufficient activation; weak virus; no contact enhancement via polycation	Verify activation. Use fresh, concentrated supernatant; add polycation; multiple spinoculation;
Good % positive but low expression	Promoter not T-cell-friendly; position effects	Try EF1α/PGK/MSC-family promoters; include an easily detectable reporter/surface tag for QC
High toxicity after transduction	Excess polycation; prolonged exposure; residual transfection reagent	Reduce polybrene; shorten exposure; promptly replace media
No improvement with higher MOI	Supernatant quality or route is limiting	Concentrate by PEG/ultracentrifuge; move to Retronectin-coated plates; split doses rather than one large hit. PEG precipitation of 24-48 h harvests is a simple approach.

## CONCLUDING REMARKS

Viral transduction is a versatile tool requiring careful optimization of cell state, vector, and culture. Read together with the preceding MiniResource on T cell preparation, this article completes a stepwise guide from isolation and activation to genetic engineering.

**Fig. 1:**
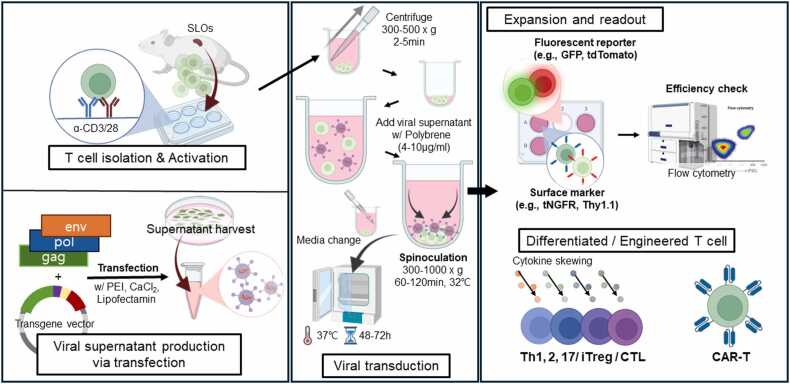
Schematic representation of the overall workflow of the viral engineering process in T cells. Isolated and activated T cells are transduced with retroviral or lentiviral vectors via spinoculation. Transgene expression can be monitored using reporter molecules (eg, GFP, tdTomato) or surface markers (eg, tNGFR, Thy1.1), allowing readout of transduction efficiency and gene delivery via flow cytometry or molecular assays.

## Funding and Support

This work was supported by the New Faculty Startup Fund from Seoul National University (Grant No. SNU-20230041 to S.S.H.), the Suh Kyungbae Foundation (Grant No. SUHF-22010039 to S.S.H.); the National Research Foundation (NRF) funded by the Ministry of Science and ICT (MSIT) (Grant Nos. RS-2021-NR059640, RS-2024-00403897, RS-2024-00398456, RS-2024-00462540, and RS-2025-15373099 to S.S.H.) (Grant No. RS-2025-25423510 to J.H.) (Grant Nos. RS-2023-00244107, RS-2024-00347015 to J.B.); the Institute of Information & Communications Technology Planning & Evaluation (IITP), funded by MSIT (Grant No. RS-2025-25463302 to S.S.H.); and the Korea–US Collaborative Research Fund (KUCRF), funded by the MSIT and the Ministry of Health & Welfare(MOHW), Republic of Korea (Grant No. RS-2025-17172968 to S.S.H.).

## Author Contributions

**Yikhyeon Seo:** Writing – review & editing, Writing – original draft, Investigation, Conceptualization. **Jimin Pak:** Writing – review & editing, Writing – original draft, Visualization, Investigation. **Jiyun Han:** Writing – review & editing, Writing – original draft, Investigation, Funding acquisition. **Joonbeom Bae:** Writing – review & editing, Funding acquisition. **Soo Seok Hwang:** Writing – review & editing, Supervision, Project administration, Funding acquisition, Conceptualization.

## Declaration of Competing Interest

The authors declare that they have no known competing financial interests or personal relationships that could have appeared to influence the work reported in this paper.
